# Targeting transcriptional regulators for treatment of anaplastic thyroid cancer

**DOI:** 10.20517/2394-4722.2021.58

**Published:** 2021-05-25

**Authors:** Woo Kyung Lee, Sheue-Yann Cheng

**Affiliations:** Laboratory of Molecular Biology, Center for Cancer Research, National Cancer Institute, National Institutes of Health, Bethesda, MD 20892, USA.

**Keywords:** Transcriptional addiction, transcription, oncogene addiction, cancer stem cell, thyroid hormone receptors, steroid receptor coactivators, BET inhibitor, anaplastic thyroid cancer

## Abstract

Dysregulation of genes perpetuates cancer progression. During carcinogenesis, cancer cells acquire dependency of aberrant transcriptional programs (known as “*transcription addiction*”) to meet the high demands for uncontrolled proliferation. The needs for particular transcription programs for cancer growth could be cancer-type-selective. The dependencies of certain transcription regulators could be exploited for therapeutic benefits. Anaplastic thyroid cancer (ATC) is an extremely aggressive human cancer for which new treatment modalities are urgently needed. Its resistance to conventional treatments and the lack of therapeutic options for improving survival might have been attributed to extensive genetic heterogeneity due to subsequent evolving genetic alterations and clonal selections during carcinogenesis. Despite this genetic complexity, mounting evidence has revealed a characteristic transcriptional addiction of ATC cells resulting in evolving diverse oncogenic signaling for cancer cell survival. The transcriptional addiction has presented a huge challenge for effective targeting as shown by the failure of previous targeted therapies. However, an emerging notion is that many different oncogenic signaling pathways activated by multiple upstream driver mutations might ultimately converge on the transcriptional responses, which would provide an opportunity to target transcriptional regulators for treatment of ATC. Here, we review the current understanding of how genetic alterations in cancer distorted the transcription program, leading to acquisition of transcriptional addiction. We also highlight recent findings from studies aiming to exploit the opportunity for targeting transcription regulators as potential therapeutics for ATC.

## INTRODUCTION

A prominent hallmark of cancer is gene dysregulation, leading to initiation of tumor development, distant metastasis, and therapeutic resistance^[1]^. Unlike normal cells, cancer cells require high levels of active transcription to develop various survival programs and maintain uncontrolled growth^[2]^. The need for high transcription to propel cancer proliferation is supported by observations of over-expressed components in transcriptional machinery in diverse cancers^[1,3–5]^. In particular, the development of aggressive and therapeutically recalcitrant tumors such as anaplastic thyroid cancer (ATC) is known to rely on characteristic patterns of gene expression (known as transcriptional addiction), despite a high level of genetic heterogeneity^[6,7]^. This transcriptional dependency requires perpetually active transcription, relying on input from transcriptional key players including chromatin regulators. Such a higher activity exhibited by transcription regulators has presented an opportunity for therapeutic intervention^[6–10]^.

ATC is a rare malignancy, but it is one of the most aggressive human solid cancers^[11]^, accounting for 1%−2% of all thyroid cancers, which are the most common endocrine malignancy^[12,13]^. ATC is extremely fast-growing and invasive, and thus most cases present as stage IV disease with distant metastasis, making most patients ineligible for surgery^[14,15]^. ATC is highly resistant to conventional therapy, and the median survival of ATC patients is less than 6 months after diagnosis^[11,16]^. Currently there are no established therapeutic options to improve overall survival of these patients^[17,18]^. American Thyroid Association guidelines and the National Comprehensive Cancer Network Clinical Practice Guidelines for ATC recommend combination therapy including surgery, chemotherapy, and/or radiotherapy to maximize clinical benefits^[16,19]^. Several new tyrosine kinase inhibitors (TKIs) are currently under evaluation in phase II clinical trials for ATC. So far only a combination therapy of dabrafenib with trametinib has been approved by the United States Food and Drug Administration for *BRAF*-mutated ATC, which was based on the limited results from a phase II clinical trial without definite evidence of benefits on survival^[20]^. Therefore, development of new effective therapeutic modalities is urgently needed.

One explanation to account for the difficulties in developing effective therapeutics for ATC is the lack of well-defined driver mutations as well as clearly elucidated molecular mechanisms underlying the carcinogenesis. ATC exhibits more genetic alterations and more extensive heterogeneity than other types of thyroid cancers^[21–23]^. The high degree of intra- or inter-tumor heterogeneity^[24,25]^ presents a huge challenge in identifying effective therapeutic targets for ATC. Nevertheless, despite this genetic complexity, in a subset of ATC, a characteristic transcriptional program frequently associated with super-enhancers emerges, resulting in constitutive activation of some oncogenes^[6,8–10,26]^. Moreover, the expression of the oncogenes that are driven by the super-enhancers has been shown to be particularly vulnerable to the effect of transcriptional inhibitors as found in other cancers^[27–31]^. Thus, the molecular modulators of these transcriptional programs, especially proteins that are important for the transcriptional control, have emerged as attractive targets for aggressive cancers such as ATC. In this review, we will briefly review the transcriptional machinery process and what is known about dysregulation of transcription in cancer development. We will then discuss how the inhibitors which could disrupt transcription could impede cancer cell survival and proliferation. We will also examine the challenges to be overcome before these inhibitors could be used for therapeutics for patients.

### General transcriptional machinery and its main regulators

**Figure 1.**
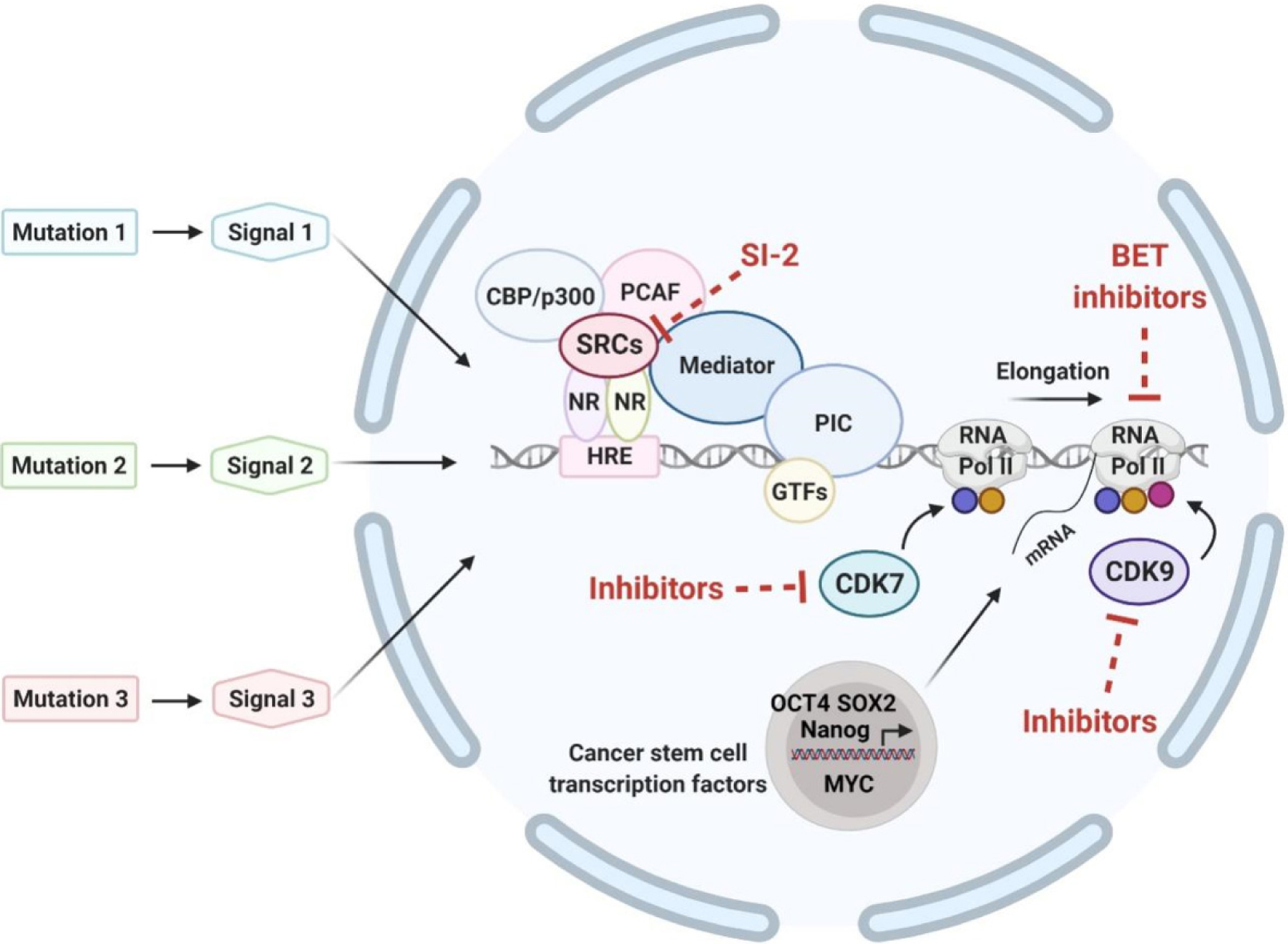
Converging of multiple oncogenic signals initiated from various upstream driver mutations in eliciting transcriptional responses. General transcription factors, mediators and RNA polymerase II assemble to form preinitiation complex to initiate transcription. The process of RNA elongation and termination is regulated by various cyclin-dependent kinases. Blue, yellow, and red circles indicate phosphorylation of the C-terminal domain of RNA Pol II at Ser 5, Ser 7, and Ser 2, respectively, by CDK7 or CDK9. Bromodomain and extra-terminal domain proteins (BET proteins; e.g., BRD4) interact with acetyl-lysines on the chromatin to activate transcription. Nuclear receptors bind to the hormone response element of target genes. Hormone/ligand-bound HRs recruit steroid hormone receptor coactivators (e.g., SRC-3) and other activators such as CBP/p300 and PCAF and together with mediator to form large complexes to further activate transcription. Mutational oncogenic upstream signals relay to converge on the transcription process to alter gene transcription output of proliferation- and differentiation-regulators to promote cancer progression. The expression of critical cancer stem cell transcription factors such as Oct4, Sox2, Nanog and MYC is known to be driven by super-enhancers on the chromatin. Sites in the transcription machinery that could be targeted by inhibitors are shown. NR: nuclear receptor; SRC: steroid hormone nuclear receptor coactivator; HRE: hormone response element; PCAF: CBP/p300-associated factor; PIC: pre-initiation complex; GTFs: general transcription factors; Pol II: polymerase II; CDK7: cyclin-dependent kinase 7; CDK9: cyclin-dependent kinase 9.

Transcription starts from the assembly of the pre-initiation complex (PIC), a complex of about 100 proteins that binds to the transcription start sites of genes and promotes DNA entry to the active site of RNA Polymerase II (RNA Pol II) for transcription initiation^[32]^ [Figure 1]. The PIC formation requires the recruitment of several general transcription factors (GTFs), which include TFIIA, TFIIB, TFIID, TFIIE, TFIIF, and TFIIH^[33]^. Promoters typically have a TATA box [TATA(A/T)A(A/T)(A/G)] located 25 base pairs upstream of the transcription start site^[34]^.

TATA binding protein (TBP), which is a subunit of TFIID, binds to the TATA box in the promoter of DNA. Subsequent recruitment of TFIIA and TFIIB stabilizes this TBP-promoter complex. TFIIB recruits RNA polymerase II and TFIIF to the promoter complex. This binding further stabilizes the RNA Pol II complex and other initiation factors on the promoter to confirm that the transcription initiation by the RNA Pol II occurs at the appropriate location^[32,35]^.

The mediator complex, which is a 23-subunit assembly, cooperatively binds with RNA Pol II and a subset of transcription factors (TFs) during the process of the PIC formation despite not binding directly to DNA sequence-specifically^[36]^. The mediator complex is recruited to promoter-enhancer regions by TFs and functions to signal the messages from the TFs to RNA Pol II, thereby enabling TF-dependent regulation of gene expression. Such communication is indispensable for transforming biological inputs from TFs to physiological responses through changes in gene expression^[37]^.

The final GTF to be recruited to the PIC is TFIIH, consisting of multiple subunits, including MAT1, cyclin-dependent kinase 7 (CDK7), its paired cyclin H, and ATP-dependent helicases (XPB and XPD)^[38]^. Following recruitment, XPB enables promoter opening for transcription to occur^[39]^, whereas CDK7-mediated phosphorylation of C-terminal domain (CTD) of RPB1, which is the largest subunit of RNA Pol II, at serine 5 induces dissociation of the mediator from the PIC, thereby leading to binding of mRNA capping enzymes that catalyze addition of the methyl-guanosine cap structure to the 5` end of nascent mRNA transcript^[40]^. CDK7 also phosphorylates TFIIE that facilitates activities of TFIIH as an ATPase and a kinase, and its phosphorylation drives the transition from transcription initiation to elongation^[41]^.

Elongation of RNA Pol II pauses 30–50 nucleotides downstream of the transcription start site. This transcriptional pause enables rapid and synchronous transcriptional activation upon release of RNA Pol II from the paused state and also functions as a check point for mRNA quality control^[42]^. The positive transcription elongation factor b (P-TEFb)/CDK9 complex is then recruited to the paused RNA Pol II and cooperates with bromodomain-containing protein 4 (BRD4) and the super elongation complex to release RNA Pol II for active transcription. While CDK7 is important for driving the initial stages of RNA Pol II elongation, CDK9 produces a fully matured elongation complex that can engage in mRNA slicing, termination, and co-transcriptionally modifying the chromatin structure^[43]^. In addition, the P-TEFb promotes the CTD phosphorylation at serine 2, a conserved marker of elongating RNA Pol II in promoting recruitment of the 3`-end processing and splicing factors for mRNA maturation^[44]^. CDK12 and CDK13 also directly contribute to the CTD phosphorylation at serine 2, transcription elongation^[45]^, splicing of pre-mRNA, and transcriptional termination^[46]^.

### Mechanisms of transcriptional dysregulation in cancer

In normal cells, cell identity is largely controlled by the action of TFs that interact with specific regions in the genome to regulate gene expression. The TFs deregulated in cancer can be subdivided into three major groups: (1) master/lineage TFs involved in organization of cell identity; (2) proliferation control TFs that can amplify transcriptional output to meet cellular demands; and (3) signaling TFs that regulate a series of machineries driven by extracellular signals. Examples of master/lineage TFs are the pluripotent OCT4, SOX2, and Nanog regulators. They function to change core regulatory circuitries and induce transcriptional activation of additional genes that are normally expressed in more embryonic states. Prime examples of proliferation control TFs are MYC and TP53, the most frequently mutated genes in human cancer. Notably, MYC can have extensive effects by amplifying the entire gene expression program^[4,47,48]^. Examples of signaling TFs are nuclear receptors including thyroid receptor (TR)^[49,50]^ and estrogen receptor (ER), STAT3, β-catenin, and NOTCH. Dysregulated signaling TFs can significantly alter the transcriptional program by binding to enhancers occupied by master TFs^[51,52]^ or aberrantly promoting super-enhancer formation^[48]^.

Besides these TFs, several cofactors play key roles in the transcriptional process. There are two classes of cofactors: the mediator complex and chromatin regulators. Mediators act as a messenger to relay signals from the TFs to chromatin regulators such as p300/CBP, MLL1–4, BRD4, JARID1A, and SWI/SNF chromatin remodeling complex. Genetic alterations of the mediator complex are frequently observed in many cancers^[53–55]^. Interestingly, few cancer-associated genetic alterations in the core RNA Pol II complex itself have been identified, suggesting that coordinated alterations of transcriptional signals upstream of RNA Pol II are more important for the neoplastic state^[56]^. Chromatin regulators are important for efficient delivery of transcriptional signals from enhancers to promoters. They function globally, and thus their dysregulation can profoundly affect the gene expression program of cancer cells^[56]^.

Recent studies have demonstrated that specific chromosomal structures play critical roles for gene regulation. The term *insulated neighborhoods* was coined to indicate that genes and their regulatory elements are typically regulated together within specific DNA loop structures. These chromosomal loop structures are bound by the CTCF protein and are co-bound with the cohesin complex^[57]^. These chromosomal structures produce specific enhancer-gene interactions that are necessary for gene activation and repression^[58–60]^. Therefore, mutations of proteins in the chromosome loop structures can profoundly affect overall gene expression. According to recent cancer genome sequencing studies, somatic mutations in the CTCF protein and the cohesion complex are frequently developed in various solid tumors, and these mutations can modify the insulated neighborhoods all over the genome, thereby rendering chromatin more accessible to oncogenic transcriptional signaling for carcinogenesis^[61,62]^.

There are classes of cis-regulatory elements that have significant roles in cancer biology: super-enhancers and insulators that form the *insulated neighborhoods*. Super-enhancers are regions of the genome comprising multiple enhancers and bind to high densities of transcriptional components to drive genes involved in maintaining specific cell identities. Cancer cells attain super-enhancers for driving the expression of oncogenes through various mechanisms^[29,63,64]^, including DNA translocation^[63]^, focal amplification^[65]^, small insertions and deletions^[66]^, and epigenomic mechanisms^[67]^. Somatic mutations in loop anchors of *insulated neighborhoods* around oncogenes also frequently occur in diverse cancers. Of note, DNA-binding motif of the CTCF protein in loop anchor regions is one of the most frequently altered TF-binding sequences in human cancer^[68]^.

In addition, transcriptional dysregulations are known to be tightly linked to epigenetic alterations, contributing to pervasive gene expression changes in cancer^[69–71]^. Epigenetic alterations are heritable and a dynamic process, altering the phenotypes by dysregulating gene expression without changes in DNA sequences. Epigenetic changes include DNA methylation, histone modifications, and the regulation of non-coding RNA^[72]^. These changes could lead to chromatin remodeling, resulting in profound changes of gene expression profiles in cancer^[72]^. This epigenetic regulation allows the genome-wide transcriptional dysregulation independent of genetic change in cancer. Interestingly, the chromatin features of cell-of-origin are known to be strong predictors for cancer mutation profiles^[73]^, suggesting chromatin alterations as critical drivers for cancer development.

### Transcriptional Regulation for Maintaining Cancer Stem Cells

For effective targeting of transcription regulators for treatment of ATC, emerging knowledge about the roles of cancer stem cells (CSCs) should be considered. The prevailing hypothesis has been that CSCs are responsible for treatment resistance and tumor relapse in aggressive cancers including ATC. CSCs are a subpopulation of cancer cells having features similar to normal embryonic stem cells (ESCs), such as self-renewal ability and pluripotency^[74]^. CSCs are endowed with the ability for self-renewal and for initiating tumors at low cell density. They can also enable a considerable portion of tumor cells to be differentiated according to tumor microenvironment. CSCs are more resistant than non-CSCs to anti-cancer therapy. Such resistance enables CSCs to evolve in the clonal selection for aggressive phenotype^[75,76]^.

Numerous studies have indicated transcriptional regulation is essential for maintaining ESC status. This transcriptional regulation is mainly mediated by pluripotency TFs such as Oct4, Sox2, Nanog, and MYC. Chromatin immunoprecipitation studies revealed extensive co-binding of Oct4, Sox2, and Nanog at many active and silent genomic target regions in ESC, indicating their role in activating other pluripotency-related factors and simultaneously suppressing lineage-specific genes^[77,78]^. While Oct4, Sox2 and Nanog cooperate with the mediator complex to recruit RNA Pol II for gene transcription^[79]^, MYC controls the transcriptional pause release of RNA Pol II through p-TEFb^[80]^ and induces the stem cell-like state by epigenetic reprograming^[81]^. Interestingly, ESC-specific genes including the pluripotency TFs and their activation targets are preferentially and frequently overexpressed in poorly differentiated aggressive human cancers^[82–84]^. Furthermore, this ESC-like gene signature is associated with poor clinical outcomes in those cancers, supporting existence of CSCs and their clinical significance^[82]^. More importantly, the genome of ESC is transcriptionally and globally hyperactive and undergoes large-scale silencing during differentiation. This transcriptional hyperactivity in ESCs is mediated by aberrant expression of the general transcriptional machinery and chromatin remodeling genes, indicating the global hyperactive transcription as a hallmark of pluripotent ESC and CSC, contributing to their plasticity^[85–89]^. Therefore, targeting transcriptional regulators would have clinical benefits for CSC depletion and re-differentiation in the treatment of ATC.

## POTENTIAL OF TRANSCRIPTIONAL REGULATORS AS BIOMARKERS AND THERAPEUTIC TARGETS IN ATC

### Thyroid hormone nuclear receptors

Thyroid hormone nuclear receptors (TRs) are members of the nuclear receptor superfamily. They are important signaling TFs to mediate biological actions of the thyroid hormone (T3) for development, growth, and metabolic homeostasis^[50,90]^. TRs generally act as ligand-dependent TFs by binding to thyroid hormone response elements (TREs) located in regulatory sites of their target genes^[91]^, but they can also control the expression of target genes that do not possess a TRE by interacting with other TFs^[92,93]^. Over the past decades, there have been major advances in understanding the physiological functions of TRs at the molecular level and, recently, their role in cancer biology. Previous studies have demonstrated that loss of heterozygosity, deletion, and reduced expression of the *THRB* gene are associated with development of diverse human cancers^[94]^. In addition, the *THRB* gene is frequently silenced through hypermethylation of its promotor region^[95–101]^ or via microRNA-mediated mechanisms^[102]^ in various cancers including thyroid cancer. These findings collectively suggest TR as a tumor suppressor. Surprisingly, a dominant-negative C-terminal frameshift mutation of TRβPV (*Thrb*^*PV/PV*^ mice) drives tumorigenesis in thyroid^[103]^, mammary^[104]^, and pituitary^[105]^ gland. These deleterious effects due to the loss of functional TRβ were clearly evident in that *TRβ*^*PV/−*^ mice^[106]^ and *Thrα1*^*−/−*^*Thrb*^*−/−*^ mice spontaneously developed metastatic follicular thyroid cancer^[107]^. That the loss of functional TRβ led to cancer development suggested that TRs broadly control transcriptional programs to suppress oncogenesis, raising the possibility that TRβ could be targeted for treatment of thyroid cancer.

This therapeutic potential of TRβ was tested in human differentiated thyroid cancer (DTC) cells. Evaluation of thyroid cancer specimens of patients and cancer cell lines showed that the expression of the *THRB* gene was suppressed through its promoter hypermethylation^[101]^. Further, the promoter hypermethylation level of the *THRB* gene was positively correlated with thyroid cancer progression. When human thyroid cancer cell lines in which the *THRB* gene was silenced through its promoter hypermethylation were treated with demethylation agents, the *THRB* gene expression was reactivated, which suppressed cancer cell proliferation and migration, and *in vivo* tumor growth in a xenograft model. These actions of the reactivated *THRB* gene occurred through suppression of the β-catenin signaling pathway in thyroid cancer cell lines^[101]^. These findings led to the direct demonstration that the exogenous expression of the *THRB* gene could suppress tumor cell proliferation and growth^[100]^. Indeed, exogenous expression of *THRB* in human follicular thyroid cancer (FTC) cells (FTC-133 and FTC-236) reduced cell proliferation and impaired cell migration through inhibition of the PI3K-AKT-mTOR pathway. Further, in xenograft tumors, the re-expressed *THRB* inhibited tumor growth and angiogenesis through suppression of vascular endothelial growth factor (VEGF) signaling pathway^[100]^. In these studies, how TRβ acted to converge these upstream signals to the nuclear transcription was not clear. The elucidation of the underlying mechanisms awaits further in-depth analysis. Nonetheless, these findings hold high promise that TRβ could be a potential therapeutic target for thyroid cancer.

### MYC and bromodomain and extra-terminal domain proteins

MYC is a master regulator of many fundamental processes such as cell cycle entry and progression, ribosome biogenesis, and metabolism. In cancer, the MYC transcriptional network is frequently overactivated through various mechanisms such as gene duplications, somatic mutations and chromosomal translocations, which increase MYC stability, thereby allowing tumor initiation and progression. *MYC* is overexpressed in more than half of all tumors and therefore has been regarded as one of the most important oncogenes in cancer^[108]^.

Uncontrolled expression of the *MYC* gene has been known to be responsible for the development and progression of ATC^[109,110]^. MYC protein is frequently upregulated in ATC tumor tissues^[110]^, and its high expression has been associated with poor clinical outcome^[111,112]^. In a mouse model of ATC, high expression of the *MYC* gene was related to thyroid cancer progression as indicated by the loss of differentiation^[113]^. In a mouse model of lung cancer, systemic inhibition of the *MYC* gene using a dominant negative *MYC* mutant resulted in complete eradication of the lung cancers^[114]^. However, so far, there has been no effective approach to directly controlling the functions of the MYC protein itself.

Chromatin remodeling through histone acetylation plays a crucial role for the transcriptional control^[115]^. The bromodomain and extra-terminal domain (BET) family of proteins, such as bromodomain-containing protein 4 (BRD4), interacts with the acetylated histones to recruit transcription activators and co-activators, and chromatin complexes to particular promoter regions^[116]^. Small-molecule inhibitors such as JQ1 selectively targeting this interaction between BET proteins and acetylated histones have been shown to potently inhibit the *MYC*-mediated transcriptional program by attenuating super-enhancers in diverse cancers^[117–122]^.

In thyroid cancer, JQ1 decreased *MYC* expression, induced cell cycle arrest, and suppressed tumor growth in a xenograft mouse model^[123,124]^. In a preclinical mouse model of ATC (*Thrb*^*PV/PV*^*Kras*^*G12D*^)^[125]^, JQ1 was found to effectively suppress *MYC* expression and attenuate *MYC*-mediated transcriptional programs, thereby inhibiting tumor growth and finally prolonging mice survival^[125]^. The efficacy of JQ1 was further tested in four cell lines originated from human ATC patients^[110]^. JQ1 markedly inhibited tumor cell proliferation through G0/G1 cell cycle arrest by suppressing *MYC* and inducing p21, p27, and RB dephosphorylation. JQ1 also could impair cancer cell invasion through attenuation of epithelial-mesenchymal transition (EMT) program. These *in vitro* findings were further confirmed by xenograft studies showing that JQ1 inhibited the size and growth rate of tumor by suppressing p21-Cyclins/CDKs-Rb-E2F signaling axis^[110]^. All these findings collectively suggested that epigenetic action of JQ1 blocking the interaction of BRD4 with histone acetyl-lysine sites across chromatin could suppress *MYC* transcription, thereby interrupting ATC progression.

**Figure 2.**
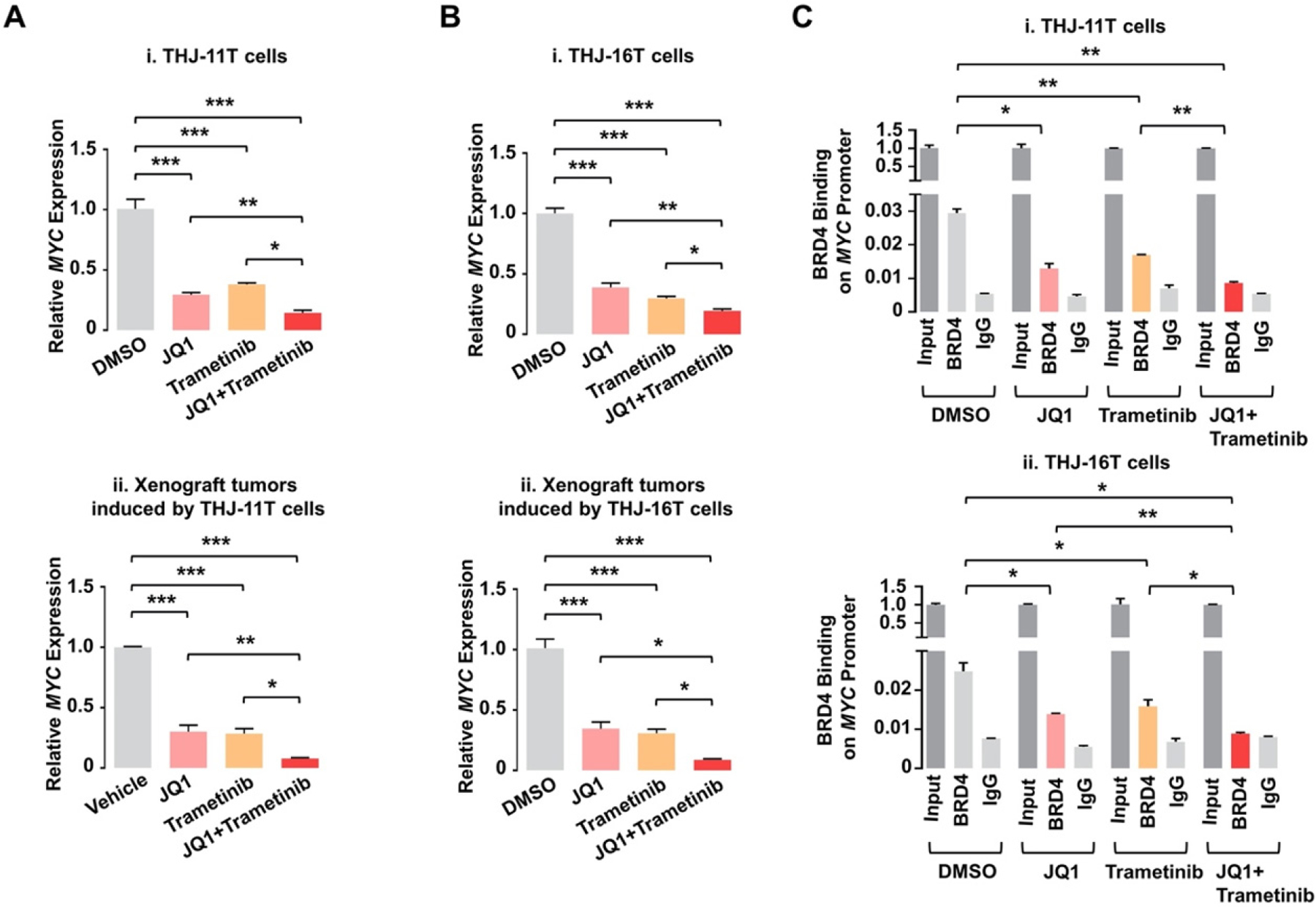
JQ1 and trametinib synergistically decrease mRNA expression of the *MYC* gene by attenuating recruitment of BRD4 to the *MYC* promoter in ATC cells and tumors. (A, B) The mRNA levels of the *MYC* gene in THJ-11T cells (A-i), −16T cells (B-i), and −11T (A-ii) and −16T xenograft tumors (B-ii). (C) Chromatin immunoprecipitation assays show BRD4 binding on the MYC promoter in THJ-11T (i) and −16T cells (ii). Significant differences were indicated by asterisks (*P < 0.05, **P < 0.01, and ***P < 0.001)^[129]^. (Permission from the authors).

Despite JQ1’s effective inhibition of tumor growth via suppression of *MYC* transcription, it showed no apparent inhibitory effects on tumor invasion and metastasis. This suggested that there were other oncogenic events to drive invasion and metastasis for the ATC progression. The MAPK-MEK signaling pathway is frequently upregulated in human ATC and is related to the ATC progression^[126–128]^. An MEK inhibitor, trametinib, was therefore used as a combined treatment with JQ1 to test this hypothesis in two human ATC cells, THJ-11T and −16T^[129]^. Remarkably, although either JQ1 alone or trametinib alone showed only partial effects, the combined treatment totally blocked proliferation of the ATC cells. Combined treatment downregulated *MYC* expression much more than each single treatment did, leading to suppression of pro-survival regulators and induction of pro-apoptotic regulators to cooperatively induce apoptosis. In xenograft studies, while each single treatment only partially inhibited growth of either THJ-11T or −16T-induced tumors, the combined treatment near completely (> 90%) blocked the tumor growth. This dramatic inhibition of tumor growth by the combined treatment occurred through synergistic suppression of *MYC*, which induced apoptotic regulators thereby markedly promoting tumor apoptosis. The underlying mechanism that the combined treatment synergistically suppressed *MYC* expression was further studied. Chromatin immunoprecipitation (ChIP) assay was used to probe the effects of JQ1 and trametinib on the binding of BRD4 to the *MYC* promoter in THJ-11T and −16T cells. As shown in Figure 2, JQ1, trametinib, and the combined treatment inhibited the BRD4 binding to the *MYC* promoter by 55%, 42%, and 69%, respectively, compared to the control (vehicle-treated cells), in THJ-11T cells [Figure 2C-i]. Similar efficacy profiles of the three treatments in the BRD4 binding suppression were observed in THJ-16T cells (46%, 36%, and 64% by JQ1, trametinib, and the combined treatment, respectively, Figure 2C-ii). These data indicated that JQ1 and trametinib functioned, at least in part, to inhibit the BRD4 binding to histone acetyl-lysine sites across the chromatin. Further, these two inhibitors could synergistically suppress *MYC* transcription via cooperative actions on chromatic modifications [Figure 2A and B].

The efficacy of combined treatment with BET and MEK inhibitors was further demonstrated by using the second generation of a BET inhibitor. A new BET inhibitor PLX51107 (PLX) has demonstrated more favorable pharmacokinetic profiles than JQ1 and other BET inhibitors^[130]^. It has been under clinical trials for various solid tumors and hematological malignancies. PD0325901 (PD) is a MEK inhibitor that has also been evaluated in clinical trials for several cancers^[131]^. PLX and PD individually could suppress proliferation of both THJ-11T and −16T cells, but together exhibited synergistic inhibition. In mouse xenografts derived from the ATC cells, the combined treatment nearly completely blocked *in vivo* tumor growth. PD effectively reduced MEK-ERK signaling, and this inhibition was further augmented by the combined treatment with PLX in the ATC cells and tumors. Notably, PLX and PD synergistically attenuate *MYC* transcription to induce p27 for the tumor suppression. They also cooperated to activate pro-apoptotic regulators to induce apoptosis. These data indicated cooperation of PLX and PD that block BRD4 binding to histone acetyl-lysine sites on the promoter of the *MYC* gene. These collaborative actions could converge to induce epigenetic modifications to suppress *MYC* transcription. The efficacy of combined treatment was clearly demonstrated by using two different sets of BET and MEK inhibitors. These findings clearly demonstrated that epigenetic modifications on chromatin is a viable and effective approach for the treatment of ATC.

### Steroid hormone nuclear receptor coactivators

The steroid hormone nuclear receptor coactivators (SRCs: SRC-1, SRC-2, and SRC-3) are important transcriptional coactivators discovered initially for the regulation of the transcriptional activity of the nuclear receptor superfamily. Subsequently, other transcription factors - including STATs, P53, RB, E2F1, hypoxia inducible factor-1 (HIF-1), Smads, and nuclear factor-κB (NF-κB) - were also found to be modulated by SRCs^[132]^. Upon ligand (hormone) binding, the ligand-bound nuclear receptors (NRs) open their coactivator-binding motifs in their ligand-binding domains and recruit SRCs to the enhancer sites of NR-target genes. SRCs further recruit other common transcriptional coactivators such as CBP/p300 and CBP/p300-associated factor (PCAF), coactivator-associated arginine methyltransferase 1 (CARM1), and protein arginine N-methyltransferase 1 (PRMT1) to the chromatin to form a NR-driven transcriptional activation complex. This protein complex uses its acetyltransferase and methyltransferase activities for chromatin remodeling to facilitate the assembly of the GTFs and RNA Pol II on the promoter for the transcriptional activation^[133–138]^ [Figure 1].

Among the SRCs, SRC-3 is the most well-studied in cancer biology. SRC-3 was initially identified as a transcriptional coactivator amplified in estrogen receptor (ER)-positive breast and ovarian cancer^[139]^. Its amplification and/or overexpression were subsequently found in diverse hormone-independent as well as - dependent cancers^[140,141]^, supporting its role for transcriptional activation of oncogenes. Supporting this notion, mice overexpressing SRC-3 developed malignant breast, pituitary, and uterine tumors through activation of the PI3K/AKT and insulin growth factor 1 (IGF-1) signaling pathway^[142]^. As mentioned in the earlier sections of this review, we generated a knock-in mutant mouse harboring a dominant negative mutant thyroid hormone receptor β mutant (*Thrb*^*PV/PV*^ mice). *Thrb*^*PV/PV*^ mice spontaneously develop aggressive FTC^[103]^. Interestingly, *Thrb*^*PV/PV*^ mice deficient in SRC-3 (*Thrb*^*PV/PV*^*Src-3*^*−/−*^) exhibit impeded thyroid cancer growth, progression and distant metastasis with a significantly increased survival, compared to *Thrb*^*PV/PV*^ mice with normal SRC-3 function (*Thrb*^*PV/PV*^*Src-3*^*+/+*^)^[143]^. These findings suggest SRC-3 as an oncogene and thus a potential therapeutic target in thyroid cancer.

**Figure 3.**
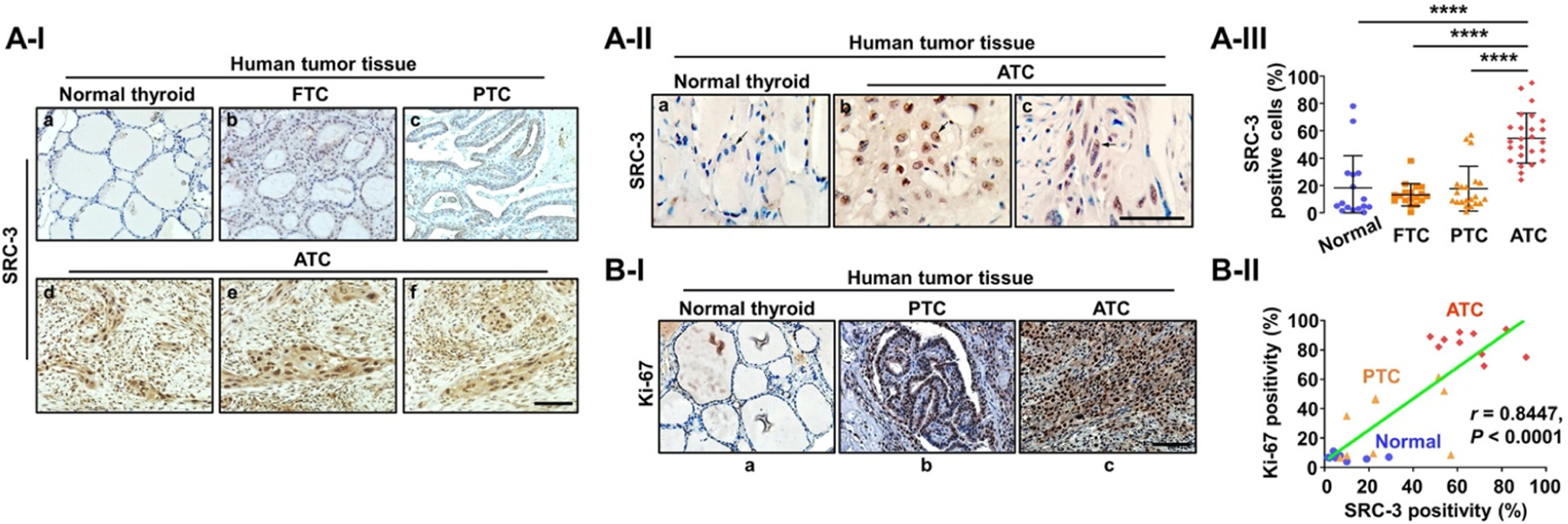
Elevated expression of SRC-3 in human ATC. (A) Immunohistochemistry (IHC) images for SRC-3 (I), their magnified images (×2) showing nuclear staining of SRC-3 (II), and quantitative analysis for the IHC results (III) in different stages of human thyroid cancer. (B) IHC images for Ki-67 (I) in normal thyroid, PTC, and ATC, and correlation plot showing strong positive relationship between SRC-3 and Ki-67 expression in ATC (II). Significant differences were indicated by asterisks (****P < 0.0001). Scale bars represent 50 μm. ATC: Anaplastic thyroid cancer; FTC: follicular thyroid cancer; PTC: papillary thyroid cancer^[147]^. (Permission from the authors).

Although transcriptional coactivators are difficult to target because of their large size and disordered structures^[144]^, a new generation SRC-3 inhibitor-2 (SI-2) was developed through cellular function-based high-throughput screening. SI-2 selectively targeted breast cancer cells through inhibition of SRC-3 transcriptional activities^[145]^. On the basis of known complex transcriptional oncogenic changes observed in ATC^[22,23,146]^ and the critical role of SRC-3 for transcriptional regulation, the expression of SRC-3 was examined during human thyroid cancer progression from normal, through DTC (FTC and PTC), to ATC^[147]^. Comparison of the SRC-3 protein abundance among human normal thyroid tissue [Figure 3A-I-a], FTC [Figure 3A-I-b], papillary thyroid cancer (PTC) [Figure 3A-I-c], and ATC [Figure 3A-I-d–f] shows that SRC-3 is clearly higher in ATCs than in normal thyroid tissues, FTC, and PTC [Figure 3A-II]. Quantitative analysis shows that 54.6% of ATC cells were positive for SRC-3 *vs.* only 18.6% of PTC cells, 13.9% of FTC cells, and 18.3% of normal thyroid cells [Figure 3A-III]. Of note, further investigation for co-expression of SRC-3 and Ki-67 (a proliferation marker), clearly demonstrated a strong positive correlation (*r* = 0.8447, *P* < 0.0001) between SRC-3 and Ki-67 expression in human ATC tissues, suggesting that hyperactive transcriptional responses through aberrant expression of SRC-3 are responsible for uncontrolled proliferation of human ATC [Figure 3B-I and II].

**Figure 4.**
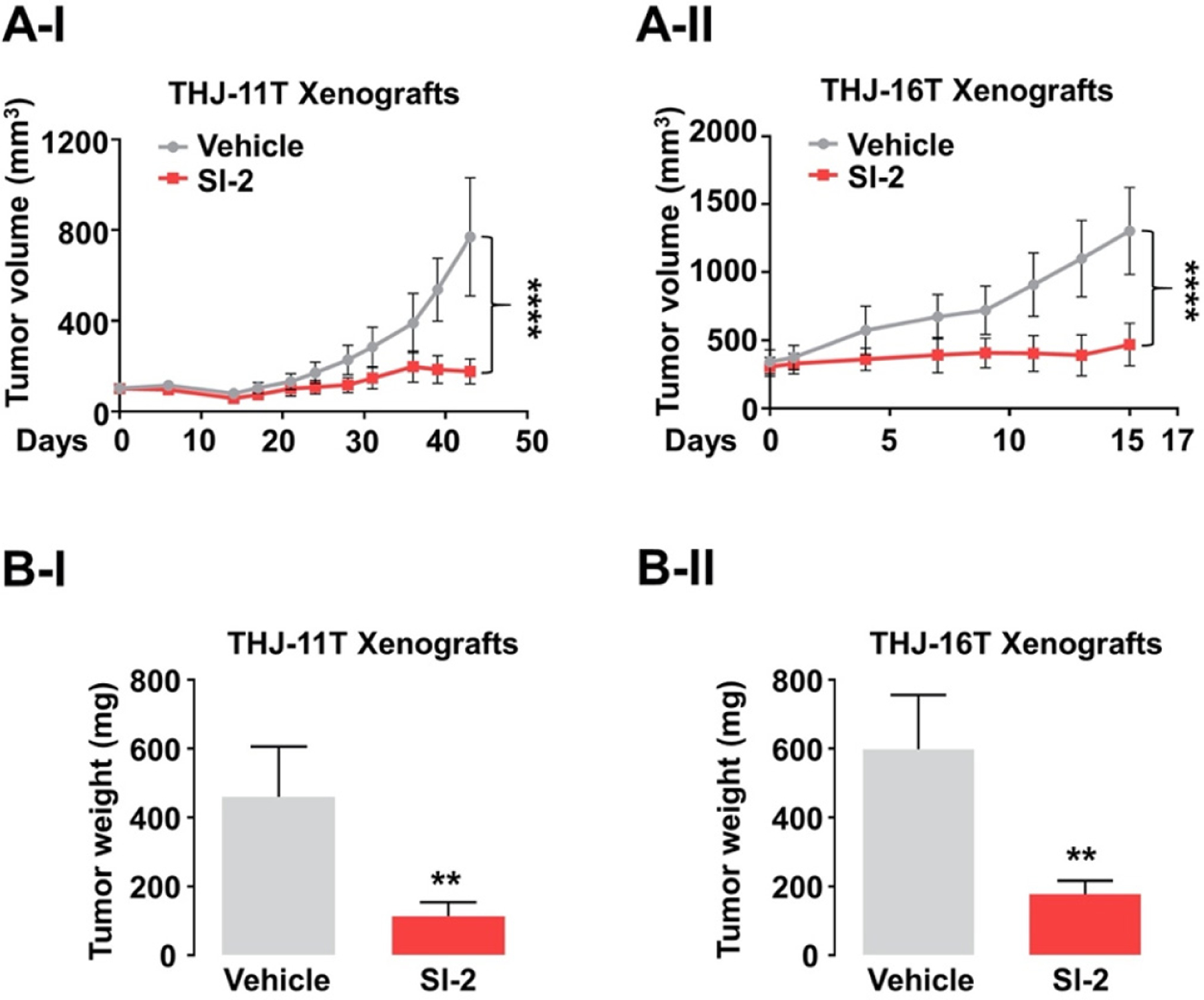
Therapeutic efficacy of SI-2 in ATC xenograft mice models. (A, B) Growth curves (A) and weight (B) of THJ-11T (I) and −16T (II) xenograft tumors treated with vehicle or SI-2. Significant differences are indicated by asterisks (**P < 0.01 and ****P < 0.0001). Data represent the mean ± SD^[147]^.

The fact that SRC-3 was both highly elevated and associated with increased proliferation provided the basis to test the efficacy of SI-2 in the treatment of ATC^[147]^. SI-2 treatment of cultured human ATC cell lines (THJ-11T and −16T) markedly suppressed tumor cell proliferation by inducing apoptosis and impeding cell cycle progression. Remarkably, growth of tumors derived from THJ-11T [Figure 4A-I] or −16T [Figure 4A-II] cells was inhibited by SI-2. The mean tumor weight was reduced by 76% and 70%, respectively, in the SI-2-treated group, compared to the vehicle-treated control group [Figure 4B-I and -II]. The inhibition of tumor growth by SI-2 was due to induction of apoptosis as evidenced by the detection of high levels of cleaved caspase 3 and pro-apoptotic regulators such as Bim in the xenograft tumors. In addition, proliferation of tumor cells was reduced as evidenced by reduced levels of Ki-67 and cyclin D1. Moreover, SI-2 blocked the activity of CSCs through inhibition of aldehyde dehydrogenase activity and expression. This observation suggested that a global transcriptional program through SRC-3 is critical for maintaining CSCs in ATC.

Finally, in-depth gene set enrichment analysis (GSEA) using The Cancer Genome Atlas Program-Thyroid Cancer (TCGA-THCA) data confirmed extensive involvement of SRC-3 in the activation of multiple oncogenic signaling pathways. The coordinated activation of 48 cancer-driver genes through SRC-3 signals poor clinical outcome in human thyroid cancer. The GSEA further indicated that this involvement of SRC-3 occurred through enrichment of genetic regions occupied by oncogenic transcription factors such as the MYC/MAX complex, NF-κB, E2F1, and ETS1^[147]^. These findings suggest that many different oncogenic signaling pathways driven by multiple upstream driver mutations assembled on the transcription responses. SRC-3 would have a critical role in the final manifestation of oncogenic transcription responses, and therefore the identification of small-molecule inhibitors such as SI-2 to target SRC-3 is a promising strategy for effective ATC treatment.

### Transcription-associated cyclin-dependent kinases

The cyclin-dependent kinases (CDKs) are the families of serine/threonine kinases that mediate fundamental cellular processes such as cell proliferation and survival. They can generally be classified into two major groups: cell cycle-related CDKs (CDK1, CDK2, CDK4, and CDK6) and transcription-associated CDKs (CDK7, CDK8, CDK9, CDK12, and CDK13). Each CDK is bound to a specific cyclin partner that guides the CDK activity. Because of their important role in cancer cell survival and growth, they have been regarded as promising therapeutic targets. Recently, CDK4/6 inhibitors have been shown to be effective in preclinical studies of multiple cancer types^[148–150]^, and impressive clinical outcomes have been demonstrated in hormone-positive breast cancer^[151,152]^. Development of small-molecule inhibitors targeting the transcription-associated CDKs has been slow, and few have entered into clinical use. Still, growing numbers of studies have shown strong efficacy of these inhibitors, particularly in a subset of cancers that exhibit the transcriptional addiction, including small cell lung cancer^[10]^, ovarian cancer^[8]^, and triple-negative breast cancer^[6]^, and T-cell acute lymphoblastic leukemia^[9]^ [Figure 1].

The efficacy of targeting the transcription-associated CDKs has also been shown in aggressive medullary thyroid cancer (MTC) and ATC. The development and progression of MTC are known to be driven by the gain of function mutations of the *RET* proto-oncogene. A super-enhancer in the intron 1 of the *RET* gene provides the sensitivity to be targeted by CDK9 inhibitors alone or with a RET kinase inhibitor^[153]^. ATC cells that exhibit super-enhancers-mediated transcription addiction were shown to be sensitive to transcription inhibition by the CDK7^[26]^ or CDK12 inhibitor^[154]^. However, intriguingly, *MYC* was not found in the list of the super-enhancer-mediated or THZ1 (CDK7 inhibitor)-sensitive cancer genes in these studies. Of note, previous findings reported that ATC cells heavily rely on MYC-driven transcriptional addiction^[110,125,155]^ and that the CDK7 inhibitor led to massive suppression of MYC-driven global transcriptional amplification^[8,10,156]^. One possible explanation for these differences among studies could be the use of different experimental models. Therefore, it would be important to develop additional experimental models that could be comprehensively analyzed and validated. Furthermore, in view of the importance of CSCs in chemoresistance and recurrence of ATC, the potential effects of inhibitors targeting the transcription-associated CDKs on CSC activity should be evaluated and its underlying molecular mechanisms elucidated. The fruitful outcome of such studies will broaden the availability of urgently needed therapeutic targets for ATC.

## CONCLUSION AND FUTURE PERSPECTIVES

ATC’s complex and heterogeneous genetic profiles with high transcriptional output enable continuous development of survival programs in the face of current targeted therapies. Early studies showed multiple upstream driver mutations to initiate carcinogenesis. More recent studies indicated that such driver mutations initiated upstream could converge to trigger transcriptional responses as evidenced by global genomic analyses. Mutations in transcriptional regulators such as components of SWI/SNF chromatin remodeling complex, histone methyltransferases, and *EIF1AX* (a key component of the translational preinitiation complex) were identified in ATC^[22,23,146]^, supporting the potential of transcriptional regulators as therapeutic targets for ATC treatment.

Indeed, as presented in this review, our studies have provided the rationale for potential clinical trials using small-molecule inhibitors such as JQ1 (BET inhibitor), PLX51107 (BET inhibitor), and SI-2 (SRC-3 inhibitor) to target the transcriptional regulators in ATC patients. Targeting other key components of the transcriptional machinery, such as chromatin regulators, the mediator, and other transcriptional coactivators, would also have profound effects on the final manifestation of oncogenic transcriptional responses. Thus, the identification of small-molecule inhibitors targeting them (or activators for TRs) is a promising strategy for effective ATC treatment.

Several important questions require further investigations to bridge the gap between preclinical studies and clinical application. Therapeutic windows for each inhibitor would have to be assessed in clinical trials because the transcriptional inhibition can affect normal as well as cancer cells. Defining the therapeutic windows for selectively targeting the cancer transcription program could avoid side effects thereby enhancing the well-being of the patients. Further, identification of transcription inhibitors which could deplete CSCs in ATC could minimize chemoresistance and recurrence. The elucidation of how the key players regulating the transcription programs lead to depleting of CSCs would certainly expand the choice of therapeutic targets for ATC to further benefit patients.

## References

[1] Bradner JE, Hnisz D, Young RA. Transcriptional addiction in cancer. Cell 2017;168:629–43. 28187285 10.1016/j.cell.2016.12.013 PMC5308559

[2] Franco HL, Kraus WL. No driver behind the wheel? Cell 2015;163:28–30. 26406367 10.1016/j.cell.2015.09.013

[3] Adhikary S, Eilers M. Transcriptional regulation and transformation by Myc proteins. Nat Rev Mol Cell Biol 2005;6:635–45. 16064138 10.1038/nrm1703

[4] Lin CY, Lovén J, Rahl PB, Transcriptional Amplification in tumor cells with elevated c-Myc. Cell 2012;151:56–67. 23021215 10.1016/j.cell.2012.08.026 PMC3462372

[5] Luo Z, Lin C, Guest E, The super elongation complex family of RNA polymerase II elongation factors: gene target specificity and transcriptional output. Mol Cell Biol 2012;32:2608–17. 22547686 10.1128/MCB.00182-12 PMC3434493

[6] Wang Y, Zhang T, Kwiatkowski N, CDK7-dependent transcriptional addiction in triple-negative breast cancer. Cell 2015;163:174–86. 26406377 10.1016/j.cell.2015.08.063 PMC4583659

[7] Zanconato F, Battilana G, Forcato M, Transcriptional addiction in cancer cells is mediated by YAP/TAZ through BRD4. Nat Med 2018;24:1599–610. 30224758 10.1038/s41591-018-0158-8 PMC6181206

[8] Zeng M, Kwiatkowski NP, Zhang T, Targeting MYC dependency in ovarian cancer through inhibition of CDK7 and CDK12/13. Elife 2018;7:e39030. 30422115 10.7554/eLife.39030 PMC6251623

[9] Kwiatkowski N, Zhang T, Rahl PB, Targeting transcription regulation in cancer with a covalent CDK7 inhibitor. Nature 2014;511:616–20. 25043025 10.1038/nature13393 PMC4244910

[10] Christensen CL, Kwiatkowski N, Abraham BJ, Targeting transcriptional addictions in small cell lung cancer with a covalent CDK7 inhibitor. Cancer cell 2014;26:909–22. 25490451 10.1016/j.ccell.2014.10.019 PMC4261156

[11] Molinaro E, Romei C, Biagini A, Anaplastic thyroid carcinoma: from clinicopathology to genetics and advanced therapies. Nat Rev Endocrinol 2017;13:644–60. 28707679 10.1038/nrendo.2017.76

[12] Lee WK, Lee J, Kim H, Peripheral location and infiltrative margin predict invasive features of papillary thyroid microcarcinoma. Eur J Endocrinol 2019;181:139–49. 31146263 10.1530/EJE-18-1025

[13] Choi JB, Lee WK, Lee SG, Long-term oncologic outcomes of papillary thyroid microcarcinoma according to the presence of clinically apparent lymph node metastasis: a large retrospective analysis of 5,348 patients. Cancer Manag Res 2018;10:2883–91. 30214283 10.2147/CMAR.S173853 PMC6118257

[14] Tuttle RM, Haugen B, Perrier ND. Updated American Joint Committee on cancer/tumor-node-metastasis staging system for differentiated and anaplastic thyroid cancer: what changed and why? Thyroid 2017;27:751–6. 28463585 10.1089/thy.2017.0102 PMC5467103

[15] Ferrari SM, Elia G, Ragusa F, Novel treatments for anaplastic thyroid carcinoma. Gland Surg 2020;9:S28–42. 32055496 10.21037/gs.2019.10.18 PMC6995904

[16] Smallridge RC, Ain KB, Asa SL, American Thyroid Association guidelines for management of patients with anaplastic thyroid cancer. Thyroid 2012;22:1104–39. 23130564 10.1089/thy.2012.0302

[17] Tiedje V, Stuschke M, Weber F, Dralle H, Moss L, Fuhrer D. Anaplastic thyroid carcinoma: review of treatment protocols. Endocr Relat Cancer 2018;25:R153–61. 29295821 10.1530/ERC-17-0435

[18] Smallridge RC. Approach to the patient with anaplastic thyroid carcinoma. J Clin Endocrinol Metab 2012;97:2566–72. 22869844 10.1210/jc.2012-1314 PMC3410281

[19] Haddad RI, Lydiatt WM, Ball DW, Anaplastic thyroid carcinoma, version 2.2015. J Natl Compr Canc Netw 2015;13:1140–50. 26358798 10.6004/jnccn.2015.0139 PMC4986600

[20] Subbiah V, Kreitman RJ, Wainberg ZA, Dabrafenib and trametinib treatment in patients with locally advanced or metastatic BRAF V600-mutant anaplastic thyroid cancer. J Clin Oncol 2018;36:7–13. 29072975 10.1200/JCO.2017.73.6785 PMC5791845

[21] Kunstman JW, Juhlin CC, Goh G, Characterization of the mutational landscape of anaplastic thyroid cancer via whole-exome sequencing. Hum Mol Genet 2015;24:2318–29. 25576899 10.1093/hmg/ddu749 PMC4380073

[22] Pozdeyev N, Gay LM, Sokol ES, Genetic analysis of 779 advanced differentiated and anaplastic thyroid cancers. Clin Cancer Res 2018;24:3059–68. 29615459 10.1158/1078-0432.CCR-18-0373 PMC6030480

[23] Yoo SK, Song YS, Lee EK, Integrative analysis of genomic and transcriptomic characteristics associated with progression of aggressive thyroid cancer. Nat Commun 2019;10:1–12. 31235699 10.1038/s41467-019-10680-5 PMC6591357

[24] Lee WK, Lee SG, Yim SH, Whole exome sequencing identifies a novel hedgehog-interacting protein g516r mutation in locally advanced papillary thyroid cancer. Int J Mol Sci 2018;19:2867. 10.3390/ijms19102867 PMC6213497 30241415

[25] Le Pennec S, Konopka T, Gacquer D, Intratumor heterogeneity and clonal evolution in an aggressive papillary thyroid cancer and matched metastases. Endocr Relat Cancer 2015;22:205–16. 25691441 10.1530/ERC-14-0351

[26] Cao X, Dang L, Zheng X, Targeting super-enhancer-driven oncogenic transcription by CDK7 inhibition in anaplastic thyroid carcinoma. Thyroid 2019;29:809–23. 30924726 10.1089/thy.2018.0550

[27] Whyte WA, Orlando DA, Hnisz D, Master transcription factors and mediator establish super-enhancers at key cell identity genes. Cell 2013;153:307–19. 23582322 10.1016/j.cell.2013.03.035 PMC3653129

[28] Lovén J, Hoke HA, Lin CY, Selective inhibition of tumor oncogenes by disruption of super-enhancers. Cell 2013;153:320–34. 23582323 10.1016/j.cell.2013.03.036 PMC3760967

[29] Chapuy B, McKeown MR, Lin CY, Discovery and characterization of super-enhancer-associated dependencies in diffuse large B cell lymphoma. Cancer cell 2013;24:777–90. 24332044 10.1016/j.ccr.2013.11.003 PMC4018722

[30] Hnisz D, Abraham BJ, Lee TI, Super-enhancers in the control of cell identity and disease. Cell 2013;155:934–47. 24119843 10.1016/j.cell.2013.09.053 PMC3841062

[31] Shi J, Whyte WA, Zepeda-Mendoza CJ, Role of SWI/SNF in acute leukemia maintenance and enhancer-mediated Myc regulation. Genes Dev 2013;27:2648–62. 24285714 10.1101/gad.232710.113 PMC3877755

[32] Sainsbury S, Bernecky C, Cramer P. Structural basis of transcription initiation by RNA polymerase II. Nat Rev Mol Cell Biol 2015;16:129–43. 25693126 10.1038/nrm3952

[33] Asturias FJ. RNA polymerase II structure, and organization of the preinitiation complex. Curr Opin Struct Biol 2004;14:121–9. 15093825 10.1016/j.sbi.2004.03.007

[34] Warfield L, Ramachandran S, Baptista T, Devys D, Tora L, Hahn S. Transcription of nearly all yeast RNA polymerase II-transcribed genes is dependent on transcription factor TFIID. Mol Cell 2017;68:118–29.e5. 28918900 10.1016/j.molcel.2017.08.014 PMC5679267

[35] Thomas MC, Chiang C-M. The general transcription machinery and general cofactors. Crit Rev Biochem Mol Biol 2006;41:105–78. 16858867 10.1080/10409230600648736

[36] Mittler G, Kremmer E, Timmers HTM, Meisterernst M. Novel critical role of a human Mediator complex for basal RNA polymerase II transcription. EMBO Rep 2001;2:808–13. 11559591 10.1093/embo-reports/kve186 PMC1084041

[37] Poss ZC, Ebmeier CC, Taatjes DJ. The Mediator complex and transcription regulation. Crit Rev Biochem Mol Biol 2013;48:575–608. 24088064 10.3109/10409238.2013.840259 PMC3852498

[38] Compe E, Egly J-M. TFIIH: when transcription met DNA repair. Nat Rev Mol Cell Biol 2012;13:343–54. 22572993 10.1038/nrm3350

[39] Moreland RJ, Tirode F, Yan Q, Conaway JW, Egly J-M, Conaway RC. A role for the TFIIH XPB DNA helicase in promoter escape by RNA polymerase II. J Biol Chem 1999;274:22127–30. 10428772 10.1074/jbc.274.32.22127

[40] Ghosh A, Shuman S, Lima CD. Structural insights to how mammalian capping enzyme reads the CTD code. Mol Cell 2011;43:299–310. 21683636 10.1016/j.molcel.2011.06.001 PMC3142331

[41] Larochelle S, Amat R, Glover-Cutter K, Cyclin-dependent kinase control of the initiation-to-elongation switch of RNA polymerase II. Nat Struct Mol Biol 2012;19:1108–15. 23064645 10.1038/nsmb.2399 PMC3746743

[42] Jonkers I, Lis JT. Getting up to speed with transcription elongation by RNA polymerase II. Nat Rev Mol Cell Biol 2015;16:167–77. 25693130 10.1038/nrm3953 PMC4782187

[43] Marshall NF, Price DH. Purification of P-TEFb, a transcription factor required for the transition into productive elongation. J Biol Chem 1995;270:12335–8. 7759473 10.1074/jbc.270.21.12335

[44] Hsin JP, Manley JL. The RNA polymerase II CTD coordinates transcription and RNA processing. Genes Dev 2012;26:2119–37. 23028141 10.1101/gad.200303.112 PMC3465734

[45] Fan Z, Devlin JR, Hogg SJ, CDK13 cooperates with CDK12 to control global RNA polymerase II processivity. Sci Adv 2020;6:eaaz5041. 32917631 10.1126/sciadv.aaz5041 PMC7190357

[46] Liang K, Gao X, Gilmore JM, Characterization of human cyclin-dependent kinase 12 (CDK12) and CDK13 complexes in C-terminal domain phosphorylation, gene transcription, and RNA processing. Mol Cell Biol 2015;35:928–38. 25561469 10.1128/MCB.01426-14 PMC4333096

[47] Nie Z, Hu G, Wei G, c-Myc is a universal amplifier of expressed genes in lymphocytes and embryonic stem cells. Cell 2012;151:68–79. 23021216 10.1016/j.cell.2012.08.033 PMC3471363

[48] Brown JD, Lin CY, Duan Q, NF-κB directs dynamic super enhancer formation in inflammation and atherogenesis. Mol Cell 2014;56:219–31. 25263595 10.1016/j.molcel.2014.08.024 PMC4224636

[49] Præstholm SM, Siersbæk MS, Nielsen R, Multiple mechanisms regulate H3 acetylation of enhancers in response to thyroid hormone. PLoS Genet 2020;16:e1008770. 32453730 10.1371/journal.pgen.1008770 PMC7274477

[50] Cheng SY, Leonard JL, Davis PJ. Molecular aspects of thyroid hormone actions. Endocr Rev 2010;31:139–70. 20051527 10.1210/er.2009-0007 PMC2852208

[51] Mullen AC, Orlando DA, Newman JJ, Master transcription factors determine cell-type-specific responses to TGF-β signaling. Cell 2011;147:565–76. 22036565 10.1016/j.cell.2011.08.050 PMC3212730

[52] Trompouki E, Bowman TV, Lawton LN, Lineage regulators direct BMP and Wnt pathways to cell-specific programs during differentiation and regeneration. Cell 2011;147:577–89. 22036566 10.1016/j.cell.2011.09.044 PMC3219441

[53] Allen BL, Taatjes DJ. The Mediator complex: a central integrator of transcription. Nat Rev Mol Cell Biol 2015;16:155–66. 25693131 10.1038/nrm3951 PMC4963239

[54] Barbieri CE, Baca SC, Lawrence MS, Exome sequencing identifies recurrent SPOP, FOXA1 and MED12 mutations in prostate cancer. Nat Genet 2012;44:685–9. 22610119 10.1038/ng.2279 PMC3673022

[55] Makinen N, Mehine M, Tolvanen J, MED12, the mediator complex subunit 12 gene, is mutated at high frequency in uterine leiomyomas. Science 2011;334:252–5. 21868628 10.1126/science.1208930

[56] Jones PA, Issa JP, Baylin S. Targeting the cancer epigenome for therapy. Nat Rev Genet 2016;17:630–41. 27629931 10.1038/nrg.2016.93

[57] Hnisz D, Day DS, Young RA. Insulated neighborhoods: structural and functional units of mammalian gene control. Cell 2016;167:1188–200. 27863240 10.1016/j.cell.2016.10.024 PMC5125522

[58] Gorkin DU, Leung D, Ren B. The 3D genome in transcriptional regulation and pluripotency. Cell stem cell 2014;14:762–75. 24905166 10.1016/j.stem.2014.05.017 PMC4107214

[59] Gibcus JH, Dekker J. The hierarchy of the 3D genome. Mol Cell 2013;49:773–82. 23473598 10.1016/j.molcel.2013.02.011 PMC3741673

[60] Phillips-Cremins JE, Corces VG. Chromatin insulators: linking genome organization to cellular function. Mol Cell 2013;50:461–74. 23706817 10.1016/j.molcel.2013.04.018 PMC3670141

[61] Lawrence MS, Stojanov P, Mermel CH, Discovery and saturation analysis of cancer genes across 21 tumour types. Nature 2014;505:495–501. 24390350 10.1038/nature12912 PMC4048962

[62] Viny AD, Ott CJ, Spitzer B, Dose-dependent role of the cohesin complex in normal and malignant hematopoiesis. J Cell Mol Med 2015;212:1819–32. 10.1084/jem.20151317 PMC4612085 26438361

[63] Drier Y, Cotton MJ, Williamson KE, An oncogenic MYB feedback loop drives alternate cell fates in adenoid cystic carcinoma. Nat Genet 2016;48:265–72. 26829750 10.1038/ng.3502 PMC4767593

[64] Tomazou EM, Sheffield NC, Schmidl C, Epigenome mapping reveals distinct modes of gene regulation and widespread enhancer reprogramming by the oncogenic fusion protein EWS-FLI1. Cell Rep 2015;10:1082–95. 25704812 10.1016/j.celrep.2015.01.042 PMC4542316

[65] Zhang X, Choi PS, Francis JM, Identification of focally amplified lineage-specific super-enhancers in human epithelial cancers. Nat Genet 2016;48:176–82. 26656844 10.1038/ng.3470 PMC4857881

[66] Mansour MR, Abraham BJ, Anders L, An oncogenic super-enhancer formed through somatic mutation of a noncoding intergenic element. Science 2014;346:1373–7. 25394790 10.1126/science.1259037 PMC4720521

[67] Nabet B, Broin PÓ, Reyes JM, Deregulation of the Ras-Erk signaling axis modulates the enhancer landscape. Cell Rep 2015;12:1300–13. 26279576 10.1016/j.celrep.2015.06.078 PMC4551578

[68] Katainen R, Dave K, Pitkänen E, CTCF/cohesin-binding sites are frequently mutated in cancer. Nat Genet 2015;47:818–21. 26053496 10.1038/ng.3335

[69] Esteller M Epigenetics in cancer. N Engl J Med 2008;358:1148–59. 18337604 10.1056/NEJMra072067

[70] Ando M, Saito Y, Xu G, Chromatin dysregulation and DNA methylation at transcription start sites associated with transcriptional repression in cancers. Nat Commun 2019;10:2188. 31097695 10.1038/s41467-019-09937-w PMC6522544

[71] Wang Z, Yin J, Zhou W, Complex impact of DNA methylation on transcriptional dysregulation across 22 human cancer types. Nucleic Acids Res 2020;48:2287–302. 32002550 10.1093/nar/gkaa041 PMC7049702

[72] Baylin SB, Jones PA. Epigenetic determinants of cancer. Cold Spring Harb Perspect Biol 2016;8:a019505. 27194046 10.1101/cshperspect.a019505 PMC5008069

[73] Polak P, Karlić R, Koren A, Cell-of-origin chromatin organization shapes the mutational landscape of cancer. Nature 2015;518:360–4. 25693567 10.1038/nature14221 PMC4405175

[74] Ayob AZ, Ramasamy TS. Cancer stem cells as key drivers of tumour progression. J Biomed Sci 2018;25:20. 29506506 10.1186/s12929-018-0426-4 PMC5838954

[75] Batlle E, Clevers H. Cancer stem cells revisited. Nat Med 2017;23:1124–34. 28985214 10.1038/nm.4409

[76] Greaves M, Maley CC. Clonal evolution in cancer. Nature 2012;481:306–13. 22258609 10.1038/nature10762 PMC3367003

[77] Loh YH, Wu Q, Chew JL, The Oct4 and Nanog transcription network regulates pluripotency in mouse embryonic stem cells. Nat Genet 2006;38:431–40. 16518401 10.1038/ng1760

[78] Boyer LA, Lee TI, Cole MF, Core transcriptional regulatory circuitry in human embryonic stem cells. Cell 2005;122:947–56. 16153702 10.1016/j.cell.2005.08.020 PMC3006442

[79] Kagey MH, Newman JJ, Bilodeau S, Mediator and cohesin connect gene expression and chromatin architecture. Nature 2010;467:430–5. 20720539 10.1038/nature09380 PMC2953795

[80] Rahl PB, Lin CY, Seila AC, c-Myc regulates transcriptional pause release. Cell 2010;141:432–45. 20434984 10.1016/j.cell.2010.03.030 PMC2864022

[81] Poli V, Fagnocchi L, Fasciani A, MYC-driven epigenetic reprogramming favors the onset of tumorigenesis by inducing a stem cell-like state. Nat Commun 2018;9:1024. 29523784 10.1038/s41467-018-03264-2 PMC5844884

[82] Ben-Porath I, Thomson MW, Carey VJ, An embryonic stem cell-like gene expression signature in poorly differentiated aggressive human tumors. Nat Genet 2008;40:499–507. 18443585 10.1038/ng.127 PMC2912221

[83] Wong DJ, Liu H, Ridky TW, Cassarino D, Segal E, Chang HY. Module map of stem cell genes guides creation of epithelial cancer stem cells. Cell Stem Cell 2008;2:333–44. 18397753 10.1016/j.stem.2008.02.009 PMC2628721

[84] Kim J, Woo AJ, Chu J, A Myc network accounts for similarities between embryonic stem and cancer cell transcription programs. Cell 2010;143:313–24. 20946988 10.1016/j.cell.2010.09.010 PMC3018841

[85] Efroni S, Duttagupta R, Cheng J, Global transcription in pluripotent embryonic stem cells. Cell Stem Cell 2008;2:437–47. 18462694 10.1016/j.stem.2008.03.021 PMC2435228

[86] Bernstein BE, Mikkelsen TS, Xie X, A bivalent chromatin structure marks key developmental genes in embryonic stem cells. Cell 2006;125:315–26. 16630819 10.1016/j.cell.2006.02.041

[87] Azuara V, Perry P, Sauer S, Chromatin signatures of pluripotent cell lines. Nat Cell Biol 2006;8:532–8. 16570078 10.1038/ncb1403

[88] Gan Q, Yoshida T, McDonald OG, Owens GK. Concise review: epigenetic mechanisms contribute to pluripotency and cell lineage determination of embryonic stem cells. Stem Cells 2007;25:2–9. 17023513 10.1634/stemcells.2006-0383

[89] Mikkelsen TS, Ku M, Jaffe DB, Genome-wide maps of chromatin state in pluripotent and lineage-committed cells. Nature 2007;448:553–60. 17603471 10.1038/nature06008 PMC2921165

[90] Lee WK, Hwang S, Kim D, Distinct features of nonthyroidal illness in critically ill patients with infectious diseases. Medicine (Baltimore) 2016;95:e3346. 27057916 10.1097/MD.0000000000003346 PMC4998832

[91] Aranda A, Pascual A. Nuclear hormone receptors and gene expression. Physiol Rev 2001;81:1269–304. 11427696 10.1152/physrev.2001.81.3.1269

[92] Méndez-Pertuz M, Sánchez-Pacheco A, Aranda A. The thyroid hormone receptor antagonizes CREB-mediated transcription. EMBO J 2003;22:3102–12. 12805224 10.1093/emboj/cdg295 PMC162147

[93] Rogatsky I, Zarember KA, Yamamoto KR. Factor recruitment and TIF2/GRIP1 corepressor activity at a collagenase-3 response element that mediates regulation by phorbol esters and hormones. EMBO J 2001;20:6071–83. 11689447 10.1093/emboj/20.21.6071 PMC125702

[94] Kim WG, Cheng SY. Thyroid hormone receptors and cancer. Biochim Biophys Acta 2013;1830:3928–36. 22507269 10.1016/j.bbagen.2012.04.002 PMC3406244

[95] Li Z, Meng ZH, Chandrasekaran R, Biallelic inactivation of the thyroid hormone receptor β1 gene in early stage breast cancer. Cancer Res 2002;62:1939–43. 11929806

[96] Iwasaki Y, Sunaga N, Tomizawa Y, Epigenetic inactivation of the thyroid hormone receptor β1 gene at 3p24. 2 in lung cancer. Ann Surg Oncol 2010;17:2222–8. 20155399 10.1245/s10434-010-0956-9

[97] Joseph B, Ji M, Liu D, Hou P, Xing M. Lack of mutations in the thyroid hormone receptor (TR) α and β genes but frequent hypermethylation of the TR β gene in differentiated thyroid tumors. J Clin Endocrinol Metab 2007;92:4766–70. 17911173 10.1210/jc.2007-0812

[98] Dunwell TL, Hesson LB, Pavlova TV, Epigenetic analysis of childhood acute lymphoblastic leukemia. Epigenetics 2009;4:185–93. 19430199 10.4161/epi.4.3.8752

[99] Hörkkö TT, Tuppurainen K, George SM, Jernvall P, Karttunen TJ, Mäkinen MJ. Thyroid hormone receptor β1 in normal colon and colorectal cancer-association with differentiation, polypoid growth type and K-ras mutations. Int J Cancer 2006;118:1653–9. 16231318 10.1002/ijc.21556

[100] Kim WG, Zhao L, Kim DW, Willingham MC, Cheng SY. Inhibition of tumorigenesis by the thyroid hormone receptor β in xenograft models. Thyroid 2014;24:260–9. 23731250 10.1089/thy.2013.0054 PMC3926148

[101] Kim WG, Zhu X, Kim DW, Zhang L, Kebebew E, Cheng SY. Reactivation of the silenced thyroid hormone receptor β gene expression delays thyroid tumor progression. Endocrinology 2013;154:25–35. 23183175 10.1210/en.2012-1728 PMC3529371

[102] Jazdzewski K, Boguslawska J, Jendrzejewski J, Thyroid hormone receptor β (THRB) is a major target gene for microRNAs deregulated in papillary thyroid carcinoma (PTC). J Clin Endocrinol Metab 2011;96:E546–53. 21159845 10.1210/jc.2010-1594 PMC3047217

[103] Suzuki H, Willingham MC, Cheng SY. Mice with a mutation in the thyroid hormone receptor β gene spontaneously develop thyroid carcinoma: a mouse model of thyroid carcinogenesis. Thyroid 2002;12:963–9. 12490073 10.1089/105072502320908295

[104] Guigon C, Kim D, Willingham M, Cheng S. Mutation of thyroid hormone receptor-β in mice predisposes to the development of mammary tumors. Oncogene 2011;30:3381–90. 21399657 10.1038/onc.2011.50 PMC3457781

[105] Furumoto H, Ying H, Chandramouli G, An unliganded thyroid hormone β receptor activates the cyclin D1/cyclin-dependent kinase/retinoblastoma/E2F pathway and induces pituitary tumorigenesis. Mol Cell Biol 2005;25:124–35. 15601836 10.1128/MCB.25.1.124-135.2005 PMC538780

[106] Kato Y, Ying H, Willingham MC, Cheng SY. A tumor suppressor role for thyroid hormone β receptor in a mouse model of thyroid carcinogenesis. Endocrinology 2004;145:4430–8. 15231697 10.1210/en.2004-0612

[107] Zhu XG, Zhao L, Willingham MC, Cheng SY. Thyroid hormone receptors are tumor suppressors in a mouse model of metastatic follicular thyroid carcinoma. Oncogene 2010;29:1909–19. 20062085 10.1038/onc.2009.476 PMC3443884

[108] Gabay M, Li Y, Felsher DW. MYC activation is a hallmark of cancer initiation and maintenance. Cold Spring Harb Perspect Med 2014;4:a014241. 24890832 10.1101/cshperspect.a014241 PMC4031954

[109] Haugen D, Akslen L, Varhaug J, Lillehaug J. Demonstration of a TGF-α-EGF-receptor autocrine loop and c-myc protein over-expression in papillary thyroid carcinomas. Int J Cancer 1993;55:37–43. 8102129 10.1002/ijc.2910550108

[110] Enomoto K, Zhu X, Park S, Targeting MYC as a therapeutic intervention for anaplastic thyroid cancer. J Clin Endocrinol Metab 2017;102:2268–80. 28368473 10.1210/jc.2016-3771 PMC5505205

[111] Terrier P, Sheng Z, Schlumberger M, Structure and expression of c-myc and c-fos proto-oncogenes in thyroid carcinomas. Br J Cancer 1988;57:43–7. 3348948 10.1038/bjc.1988.6 PMC2246694

[112] Romano M, Grattone M, Karner M, Relationship between the level of c-myc mRNA and histologic aggressiveness in thyroid tumors. Horm Res Paediatr 1993;39:161–5. 10.1159/000182718 8262479

[113] Zhu X, Zhao L, Park JW, Willingham MC, Cheng SY. Synergistic signaling of KRAS and thyroid hormone receptor β mutants promotes undifferentiated thyroid cancer through MYC up-regulation. Neoplasia 2014;16:757–69. 25246276 10.1016/j.neo.2014.08.003 PMC4234871

[114] Soucek L, Whitfield JR, Sodir NM, Inhibition of Myc family proteins eradicates KRas-driven lung cancer in mice. Genes Dev 2013;27:504–13. 23475959 10.1101/gad.205542.112 PMC3605464

[115] Kouzarides T Chromatin modifications and their function. Cell 2007;128:693–705. 17320507 10.1016/j.cell.2007.02.005

[116] Filippakopoulos P, Picaud S, Mangos M, Histone recognition and large-scale structural analysis of the human bromodomain family. Cell 2012;149:214–31. 22464331 10.1016/j.cell.2012.02.013 PMC3326523

[117] Mertz JA, Conery AR, Bryant BM, Targeting MYC dependence in cancer by inhibiting BET bromodomains. Proc Natl Acad Sci U S A 2011;108:16669–74. 21949397 10.1073/pnas.1108190108 PMC3189078

[118] Delmore JE, Issa GC, Lemieux ME, BET bromodomain inhibition as a therapeutic strategy to target c-Myc. Cell 2011;146:904–17. 21889194 10.1016/j.cell.2011.08.017 PMC3187920

[119] Bian B, Bigonnet M, Gayet O, Gene expression profiling of patient-derived pancreatic cancer xenografts predicts sensitivity to the BET bromodomain inhibitor JQ 1: implications for individualized medicine efforts. EMBO Mol Med 2017;9:482–97. 28275007 10.15252/emmm.201606975 PMC5376755

[120] Li N, Yang L, Qi XK, BET bromodomain inhibitor JQ1 preferentially suppresses EBV-positive nasopharyngeal carcinoma cells partially through repressing c-Myc. Cell Death Dis 2018;9:761. 29988031 10.1038/s41419-018-0789-1 PMC6037792

[121] Shao Q, Kannan A, Lin Z, Stack BC, Suen JY, Gao L. BET protein inhibitor JQ1 attenuates Myc-amplified MCC tumor growth in vivo. Cancer Res 2014;74:7090–102. 25277525 10.1158/0008-5472.CAN-14-0305 PMC4322674

[122] Baratta MG, Schinzel AC, Zwang Y, An in-tumor genetic screen reveals that the BET bromodomain protein, BRD4, is a potential therapeutic target in ovarian carcinoma. Proc Natl Acad Sci U S A 2015;112:232–7. 25535366 10.1073/pnas.1422165112 PMC4291641

[123] Gao X, Wu X, Zhang X, Inhibition of BRD4 suppresses tumor growth and enhances iodine uptake in thyroid cancer. Biochem Biophys Res Commun 2016;469:679–85. 26707881 10.1016/j.bbrc.2015.12.008

[124] Mio C, Conzatti K, Baldan F, BET bromodomain inhibitor JQ1 modulates microRNA expression in thyroid cancer cells. Oncol Rep 2018;39:582–8. 29251329 10.3892/or.2017.6152

[125] Zhu X, Enomoto K, Zhao L, Bromodomain and extraterminal protein inhibitor JQ1 suppresses thyroid tumor growth in a mouse model. Clin Cancer Res 2017;23:430–40. 27440272 10.1158/1078-0432.CCR-16-0914 PMC5241246

[126] McFadden DG, Vernon A, Santiago PM, p53 constrains progression to anaplastic thyroid carcinoma in a Braf-mutant mouse model of papillary thyroid cancer. Proc Natl Acad Sci U S A 2014;111:E1600–9. 24711431 10.1073/pnas.1404357111 PMC4000830

[127] Zaballos MA, Santisteban P. Key signaling pathways in thyroid cancer. J Endocrinol 2017;235:R43–61. 28838947 10.1530/JOE-17-0266

[128] Naoum GE, Morkos M, Kim B, Arafat W. Novel targeted therapies and immunotherapy for advanced thyroid cancers. Mol Cancer 2018;17:51. 29455653 10.1186/s12943-018-0786-0 PMC5817719

[129] Zhu X, Holmsen E, Park S, Willingham MC, Qi J, Cheng SY. Synergistic effects of BET and MEK inhibitors promote regression of anaplastic thyroid tumors. Oncotarget 2018;9:35408–21. 30459933 10.18632/oncotarget.26253 PMC6226043

[130] Ozer HG, El-Gamal D, Powell B, BRD4 profiling identifies critical chronic lymphocytic leukemia oncogenic circuits and reveals sensitivity to PLX51107, a novel structurally distinct BET inhibitor. Cancer Discov 2018;8:458–77. 29386193 10.1158/2159-8290.CD-17-0902 PMC5882533

[131] Barrett SD, Bridges AJ, Dudley DT, The discovery of the benzhydroxamate MEK inhibitors CI-1040 and PD 0325901. Bioorg Med Chem Lett 2008;18:6501–4. 18952427 10.1016/j.bmcl.2008.10.054

[132] Wang L, Lonard DM, O’Malley BW. The role of steroid receptor coactivators in hormone dependent cancers and their potential as therapeutic targets. Horm Cancer 2016;7:229–35. 27125199 10.1007/s12672-016-0261-6 PMC4930410

[133] Yao TP, Ku G, Zhou N, Scully R, Livingston DM. The nuclear hormone receptor coactivator SRC-1 is a specific target of p300. Proc Natl Acad Sci U S A 1996;93:10626–31. 8855229 10.1073/pnas.93.20.10626 PMC38204

[134] Anafi M, Yang YF, Barlev NA, GCN5 and ADA adaptor proteins regulate triiodothyronine/GRIP1 and SRC-1 coactivator-dependent gene activation by the human thyroid hormone receptor. Mol Endocrinol 2000;14:718–32. 10809234 10.1210/mend.14.5.0457

[135] Brown K, Chen Y, Underhill TM, Mymryk JS, Torchia J. The coactivator p/CIP/SRC-3 facilitates retinoic acid receptor signaling via recruitment of GCN5. J Biol Chem 2003;278:39402–12. 12885766 10.1074/jbc.M307832200

[136] Koh SS, Chen D, Lee YH, Stallcup MR. Synergistic enhancement of nuclear receptor function by p160 coactivators and two coactivators with protein methyltransferase activities. J Biol Chem 2001;276:1089–98. 11050077 10.1074/jbc.M004228200

[137] Spencer TE, Jenster G, Burcin MM, Steroid receptor coactivator-1 is a histone acetyltransferase. Nature 1997;389:194–8. 9296499 10.1038/38304

[138] Zhang H, Yi X, Sun X, Differential gene regulation by the SRC family of coactivators. Genes Dev 2004;18:1753–65. 15256502 10.1101/gad.1194704 PMC478195

[139] Anzick SL, Kononen J, Walker RL, AIB1, a steroid receptor coactivator amplified in breast and ovarian cancer. Science 1997;277:965–8. 9252329 10.1126/science.277.5328.965

[140] Yan J, Tsai SY, Tsai MJ. SRC-3/AIB1: transcriptional coactivator in oncogenesis. Acta Pharmacol Sin 2006;27:387–94. 16539836 10.1111/j.1745-7254.2006.00315.x

[141] Xu J, Wu RC, O’Malley BW. Normal and cancer-related functions of the p160 steroid receptor co-activator (SRC) family. Nat Rev Cancer 2009;9:615–30. 19701241 10.1038/nrc2695 PMC2908510

[142] Torres-Arzayus MI, Font de Mora J, Yuan J, High tumor incidence and activation of the PI3K/AKT pathway in transgenic mice define AIB1 as an oncogene. Cancer Cell 2004;6:263–74. 15380517 10.1016/j.ccr.2004.06.027

[143] Ying H, Willingham M, Cheng S. The steroid receptor coactivator-3 is a tumor promoter in a mouse model of thyroid cancer. Oncogene 2008;27:823–30. 17653082 10.1038/sj.onc.1210680

[144] Lonard DM, O’malley BW. Nuclear receptor coregulators: modulators of pathology and therapeutic targets. Nat Rev Endocrinol 2012;8:598–604. 22733267 10.1038/nrendo.2012.100 PMC3564250

[145] Song X, Chen J, Zhao M, Development of potent small-molecule inhibitors to drug the undruggable steroid receptor coactivator-3. Proc Natl Acad Sci U S A 2016;113:4970–5. 27084884 10.1073/pnas.1604274113 PMC4983835

[146] Landa I, Ibrahimpasic T, Boucai L, Genomic and transcriptomic hallmarks of poorly differentiated and anaplastic thyroid cancers. J Clin Invest 2016;126:1052–66. 26878173 10.1172/JCI85271 PMC4767360

[147] Lee WK, Kim WG, Fozzatti L, Steroid receptor coactivator-3 as a target for anaplastic thyroid cancer. Endocr Relat Cancer 2020;27:209–20. 31977311 10.1530/ERC-19-0482 PMC7326649

[148] Lee HJ, Lee WK, Kang CW, Ku CR, Cho YH, Lee EJ. A selective cyclin-dependent kinase 4, 6 dual inhibitor, Ribociclib (LEE011) inhibits cell proliferation and induces apoptosis in aggressive thyroid cancer. Cancer Lett 2018;417:131–40. 29306020 10.1016/j.canlet.2017.12.037

[149] Rader J, Russell MR, Hart LS, Dual CDK4/CDK6 inhibition induces cell-cycle arrest and senescence in neuroblastoma. Clin Cancer Res 2013;19:6173–82. 24045179 10.1158/1078-0432.CCR-13-1675 PMC3844928

[150] Zhang YX, Sicinska E, Czaplinski JT, Antiproliferative effects of CDK4/6 inhibition in CDK4-amplified human liposarcoma in vitro and in vivo. Mol Cancer Ther 2014;13:2184–93. 25028469 10.1158/1535-7163.MCT-14-0387

[151] Finn RS, Martin M, Rugo HS, Palbociclib and letrozole in advanced breast cancer. N Engl J Med 2016;375:1925–36. 27959613 10.1056/NEJMoa1607303

[152] Hortobagyi GN, Stemmer SM, Burris HA, Ribociclib as first-line therapy for HR-positive, advanced breast cancer. N Engl J Med 2016;375:1738–48. 27717303 10.1056/NEJMoa1609709

[153] Valenciaga A, Saji M, Yu L, Transcriptional targeting of oncogene addiction in medullary thyroid cancer. JCI Insight 2018;3:e122225. 10.1172/jci.insight.122225 PMC6141185 30135308

[154] Geng M, Yang Y, Cao X, Dang L, Zhang T, Zhang L. Targeting CDK12-mediated transcription regulation in anaplastic thyroid carcinoma. Biochem Biophys Res Commun 2019;520:544–50. 31615655 10.1016/j.bbrc.2019.10.052

[155] Zhu X, Park S, Lee WK, Cheng SY. Potentiated anti-tumor effects of BETi by MEKi in anaplastic thyroid cancer. Endocr Relat Cancer 2019;26:739–50. 31272080 10.1530/ERC-19-0107 PMC6938575

[156] Chipumuro E, Marco E, Christensen CL, CDK7 inhibition suppresses super-enhancer-linked oncogenic transcription in MYCN-driven cancer. Cell 2014;159:1126–39. 25416950 10.1016/j.cell.2014.10.024 PMC4243043

